# Risk factors for community-acquired acute kidney injury in patients with and without chronic kidney injury and impact of its initial management on prognosis: a prospective observational study

**DOI:** 10.1186/s12882-017-0792-2

**Published:** 2017-12-29

**Authors:** Fabien Stucker, Belen Ponte, Victoria De la Fuente, Cyrielle Alves, Olivier Rutschmann, Sebastian Carballo, Nicolas Vuilleumier, Pierre-Yves Martin, Thomas Perneger, Patrick Saudan

**Affiliations:** 1 Nephrology Unit, Hôpital de la Providence, Neuchâtel, Switzerland; 2 Nephrology Unit, Department of Medical Specialties, Geneva University Hospitals, 4 rue Gabrielle Perret-Gentil, 1211 Geneva, Switzerland; 3 Department of General Internal Medicine, Geneva University Hospitals, Geneva, Switzerland; 4 Emergency Unit, Geneva University Hospitals, Geneva, Switzerland; 5 Department of Genetics and Laboratory Medicine, Geneva University Hospitals, Geneva, Switzerland; 6 Division of Clinical Epidemiology, Geneva University Hospitals, Geneva, Switzerland

**Keywords:** Community-acquired, Acute kidney injury, Chronic kidney injury, Prognosis

## Abstract

**Background:**

We aimed to describe clinical characteristics of patients with community-acquired acute kidney injury (CA-AKI), the effectiveness of initial management of CA-AKI, its prognosis and the impact of medication on its occurrence in patients with previous chronic kidney injury (CKI).

**Methods:**

We undertook a prospective observational study within the Emergency Department (ED) of a University Hospital, screening for any patient >16 years admitted with an eGFR <60 ml/mn/1.73 m^2^ and a rise in serum creatinine as compared to previous values. Patients’ medical files were reviewed by a panel of nephrologists in the subsequent days and at one and three-years follow-up.

**Results:**

From May 1st to June 21st 2013, there were 8464 admissions in the ED, of which 653 had an eGFR <60 ml/mn/1.73 m^2^. Of these, 352 had previous CKI, 341 had CA-AKI, and 104 had CA-ACKI (community-acquired acute on chronic kidney injury). Occurrence of superimposed CA-AKI in CKI patients was associated with male gender and with use of diuretics, but not with use of ARBs or ACEIs.

Adequate management of CA-AKI defined as identification, diagnostic procedures and therapeutic intervention within 24 h, was recorded in 45% of the cases and was not associated with improved outcomes.

Three-year mortality was 21 and 48% in CKI and CA-ACKI patients respectively, and 40% in patients with only CA-AKI (*p* < 0.001). Mortality was significantly associated with age, hypertension, ischemic heart disease and CA-AKI.

Progression of renal insufficiency was associated with male gender and age.

**Conclusions:**

CA-AKI is more frequently encountered in male patients and those treated with diuretics and is an independent risk factor for long-term mortality. Its initial adequate management failed to improve outcomes.

**Electronic supplementary material:**

The online version of this article (10.1186/s12882-017-0792-2) contains supplementary material, which is available to authorized users.

## Background

Acute Kidney Injury (AKI) is a frequent disorder with an incidence of 400 per 100′000 persons per year in community-based populations [1]. AKI has been associated with increased mortality and progression to CKI [2], and there is increased awareness of its ominous long-term prognosis [3]. Most studies, however, have focused on hospital-acquired AKI (HA-AKI) and very few studies have analyzed the characteristics and outcomes of patients with community-acquired acute kidney injury (CA-AKI) despite its occurrence in 1% of hospital admissions [4, 5]. A prospective Spanish study demonstrated that 52% of AKI cases were HA-AKI, without specifying whether characteristics and outcomes differed from CA-AKI [6]. A recent North-American retrospective cohort study showed that patients developing HA-AKI had more comorbidities, higher mortality rate and were more prone to need intensive care unit (ICU) care than patients with CA-AKI [7]. Another retrospective study from the United Kingdom compared the epidemiology and outcomes of patients with CA-AKI to those with HA-AKI and also found that the latter had a grimmer prognosis in spite of similar risk factors [8].

An additional issue is that early recognition and management in the Emergency Department (ED) of patients with CA-AKI may have a positive impact on prognosis. In an intensive care setting, a delayed nephrology consultation was associated with higher mortality [9, 10]. In HA-AKI patients, early involvement of a nephrology team resulted in a lower rate of creatinine increase [11]. However, in septic patients, early goal-directed therapy failed to reduce the incidence of AKI [12]. Since the implementation of Kidney Disease Improving Global outcomes (KDIGO) guidelines to diagnose and grade AKI, no study has evaluated the impact of its early recognition and management on subsequent outcomes. There is indeed a lack of data to assess the impact of early AKI management on mortality, length of hospital stay, need of renal replacement therapy (RRT), or subsequent kidney function.

We therefore aimed to better define the characteristics and prognosis of CA-AKI in patients with and without CKI, the impact of CA-AKI recognition and management within the first 24 h, and the long-term outcomes of these patients compared to patients with stable CKI.

## Methods

### Study design and subjects

We conducted a prospective observational study within the ED of Geneva University Hospitals, an urban primary and tertiary care hospital admitting 64,000 patients/yr. The study was approved by our local Ethics committee and was supported by the PRD (Research and Development Projects) program of the Geneva University Hospitals.

Over 8 weeks, patients >16 years admitted to the ED and presenting with an eGFR <60 ml/min/1.73 m^2^ were identified with the help of the Hospital computer database. GFR was estimated by the MDRD equation and the laboratory provided a quick display for those having less than 60 ml/min/1.73 m.^2^The files of screened patients were reviewed by 2 nephrologists (P.S, B.P and F.S), or 3 in case of disagreement between 2 of them. Those patients without an acute rise of creatinine were classified as stable CKI.

### Definitions

AKI was defined as an increase in serum creatinine values compared to baseline values, according to KDIGO AKI criteria: 1) an increase ≥26.5 μmol from baseline serum creatinine values, or 2) ≥ 1.5× from baseline serum creatinine values, or 3) an urine volume < 0.5 ml/kg/h for 6 h. Definition of reference baseline serum creatinine value in this study was the last previous available creatinine value within the preceding 12 months or, taking into account that some individuals were outpatients with CA-AKI and no recent or previous creatinine values, the lowest creatinine value within the next 28 days after AKI. Baseline serum creatinine could be ascertained prior to AKI in 80% of our patients. As urine output was recorded in only 10% of the ED files, this criterion was not used for AKI assessment.

Etiologies of AKI were classified prerenal, renal and postrenal based on clinical examination, renal ultrasound and urinalysis results. Renal recovery was defined as a return to within 20% of baseline creatinine within the following 90 days according to the UK renal association definition [13, 14]. Patients with an eGFR ≥60 ml/mn based on a blood test sampled beyond 90 days post CA-AKI were classified within the recovery category.

### Assessment of potentially nephrotoxic drugs

Intake of non-steroidal anti-inflammatory drugs (NSAIDs), Antibiotics, angiotensin-receptor blockers **(**ARBs), angiotensin-converting enzyme inhibitors (ACEIs) and diuretics were recorded on ED admission. ARBs, ACEIs and diuretics used in these patients were classified between low and medium-high doses, according to manufacturer’s recommended dosage for hypertension (Additional file 1: Table S1).

### Indicators of adequate AKI management

The panel of nephrologists analyzed the management of AKI during the first 24 h after ED admission, using three quality of care criteria: 1) identification and mention of AKI in the patients’ file, 2) an adequate diagnostic procedure (renal ultrasound, urinary microscopy and chemistry) and 3) a subsequent appropriate therapeutic intervention, such as correction of hypovolemia, management of sepsis, or drainage of urinary tract obstruction. Adequate management was defined as the presence of all 3 quality criteria.

### Outcomes

The same panel reviewed the hospital computer database and the Geneva civil registry at one-year follow-up and recorded the specified outcomes: hospitalization days, need for renal replacement therapy (RRT), intensive care unit (ICU) stay, survival and most recent renal serum creatinine values. The last available serum creatinine value was retrieved either in our hospital computer database or in private practitioners to determine the evolution of renal function. Kidney disease progression was based on a decline in GFR category according to KDIGO guidelines in patients with at least one-year follow-up. The Geneva civil registry was reviewed at one-year and three-year follow-up to determine survival.

### Statistical analysis

All categorical variables are presented as frequencies and percentages and all continuous variables with a symmetric distribution as mean ± SD. Skewed variables (e.g., length of stay) are expressed as median and inter-quartile range. A two-sided *p* value <0.05 was considered as significant. Statistical analysis was implemented using SPSS software, version 18 (SPSS, Inc.; Chicago, IL).

Parametric and non-parametric tests were used to compare baseline characteristics and main outcomes in patients with CA-AKI (hospitalization days, need for renal replacement therapy and death) between the 2 groups of quality criteria (3 versus ≤2 criteria reached). We used multiple logistic regression to determine factors associated with adequate AKI management in the ED, defined as the compliance of 3 quality criteria.

We used the Cox proportional hazard model to compare survival in patients with stable CKI and in patients with CA-AKI and CA-ACKI, unadjusted and adjusted for age, gender, AKI, diabetes, previous CKI and heart failure. We used linear regression and binary logistic regression for a similar analysis of length of stay, total hospital days, renal recovery within 90 days of follow-up in patients with CA-AKI and renal function decline in the 3 groups within 3 years of follow-up.

## Results

During the 8 weeks of the screening period, 8464 patients were admitted in the ED of whom 653 had an eGFR <60 ml/mn/1.73 m^2^. We excluded 64 patients: 7 were already on chronic dialysis, 54 with a ≤ 26.5 μmol/L transient rise in serum creatinine and return within 24 h to an eGFR >60 ml/mn/1.73 m^2^ and 3 patients had two admissions during the screening period. Finally, the panel of nephrologists identified 248 patients with stable CKI, 104 with CA-ACKI and 237 with CA-AKI. Table 1 shows the main characteristics of these 589 patients comparing CKI, CA-AK and CA-ACKI patients. Of note, they were predominantly elderly, with a high prevalence of hypertensive disease. ACEIs, ARBs and diuretics doses were similar in the 3 groups (Table 1). Among the 341 patients with CA-AKI and according to the chart review by the panel of nephrologists, prerenal causes were principally due to hypovolemia and heart failure.

**Table 1:** Demographics, clinical, laboratory data, medication data on admission

Patient characteristics	CKI only (*n* = 238)	CA-ACKI (*n* = 104)	CA-AKI only (*n* = 237	*p*
Mean age (yr) ± SD Median (yr) + IQR	79.7 ± 11.4 82 (74–88)	79.5 ± 12.8 83 (73–89)	72.8 ± 15.4 76 (64–84)	0.001
Male gender (%)	47	61	57	0.02
Diabetes (%)	21	29	18	0.08
Hypertension (%)	57	62	52	0.23
Coronaropathy (%)	28	30	21	0.12
Cardiac Insufficiency (%)	21	19	12	0.03
Cirrhosis (%)	1	3	2	0.54
Cancer (%)	8	9	14	0.09
Infection (%)	4	7	11	0.01
AKI characteristics				
Medical/Surgical/trauma (%)	81/11/8	81/11/9	85/10/5	0.67
Stage 1/2/3 (%)		42/41/16	79/17/4	0.001
Prerenal/renal/postrenal/mixed (%)		67/14/7/12	81/4/6/9	0.006
Mean P-Creatinine (μmol/L) ± SD	125 ± 55	270 ± 253	155 ± 96	0.001
eGFR (ml/mn) ± SD	46 ± 11	27 ± 12	41 ± 13	0.001
Nephrotoxic drugs				
RAA blockers (%)	47	48	44	0.80
ACEIs (%)	24	16	15	0.02
*Low/medium-high doses (%)*	*32/68*	*24/76*	*43/57*	*0.33*
ARBs(%)	28	34	23	0.08
*Low/medium-high doses (%)*	*26/74*	*37/63*	*17/83*	*0.07*
Diuretics (%)	40	53	35	0.008
*Low/medium-high doses (%)*	*73/27*	*50/50*	*61/39*	*0.02*
RAA blockers + diuretics (%)	25	24	26	0.99
NSAIDs (%)	3	5	6	0.37
Antibiotics (%)	2	1	6	0.04

Risk factors for CA-ACKI in the 352 CKI patients were male gender (OR: 1.71, 95% CI: 1.04-2.81, *p* = 0.03) and diuretics use. ARBs use was significantly associated with CA-AKI in univariate analysis, but not in multivariate analysis (Table 2).

**Table 2:** Occurrence of CA-AKI and association with RABs and diuretics by logistic regression in CKI patients (*n* = 352)

	CA-AKI	
	Unadjusted HR (95% CI)	Adjusted HR (95% CI)
RABs	1.03 (0.65 – 1.64) *p* = 0.89	0.85 (0.51 – 1.41)^a^ *p* = 0.53
ARBs	1.74 (1.05 – 2.88) *p* = 0.03	1.53 (0.88 – 2.66)^b^ *p* = 0.13
ACEIs	0.61 (0.34 – 1.11) *p* = 0.11	0.58 (0.30 – 1.11)^b^ *p* = 0.10
Diuretics	1.69 (1.07 – 2.68) *p* = 0.02	1.82 (1.09 – 3.04)^b^ *p* = 0.02

^a^Adjusted for age, gender, presence of diabetes, HTN, cardiac insufficiency, coronaropathy, neoplasia, infection, and diuretics

^b^Adjusted for age, gender, presence of diabetes, HTN, cardiac insufficiency, coronaropathy, neoplasia, infection, ARBs, ACEIs and diuretics

Regarding AKI management, AKI was mentioned in 53.1% (*n* = 182) of the medical records within the ED. The cases with the most severe kidney injury were more likely to be identified and recorded in the emergency file by the physician in charge: stage I 46%, stage II 74% and stage III 88%. An appropriate diagnosis work-up was done in 57% of the patients and an adequate intervention in 73% of the cases. Of note, only 11 requests (3%) for a renal consultation were made within the ED.

Overall, 44.6% (152/341) of the patients with CA-AKI were adequately managed in the ED. Age, gender, previous comorbidities such as diabetes, hypertension and heart failure were not associated with the rate of adequate management (Table 3). In multivariate analysis, presence of underlying CKI was just associated with lower management adequacy. AKI severity was associated with an increase in management adequacy. Adequate AKI management had no impact on ICU stay, need for RRT, or 1-year mortality, and was not associated with more renal recovery in the 313 patients who had a minimum 28-day follow-up (Table 4). Adequate AKI management was associated with a significantly longer hospital stay and more hospitalization days within the subsequent year. This trend subsisted after adjustment for age, gender, presence of diabetes, CKI and heart failure (Table 5). Results were also similar when each quality criteria were analyzed separately (data not shown).

**Table 3:** Variables associated with adequate management of CA-AKI (all 3 criteria satisfied) in 341 patients

	Adequate n (%)	*P* value	Univariate			Multivariate		
			OR	95% CI	*P* value	OR	95% CI	*P* value
Age groups		0.57^a^			0.57^a^			0.38^a^
25–64	32 (42.7)		1.00	–		1.00	–	
65–74	28 (43.8)		1.04	0.53 – 2.05		0.76	0.36 – 1.62	
75–84	44 (44.0)		1.06	0.58 – 1.93		1.27	0.66 – 2.46	
85–103	48 (47.1)		1.19	0.66 – 2.18		1.19	0.60 – 2.34	
Sex		0.88			0.88			0.84
Women	64 (45.1)		1.00	–		1.00	–	
Men	88 (44.2)		0.97	0.63-1.49		1.05	0.65-1.70	
AKI stage		<0.001^a^			<0.001			<0.001
I	79 (34.3)		1.00	–		1.00	–	
II	51 (60.7)		2.95	1.76 – 4.95		3.88	2.17 – 6.93	
III	22 (84.6)		10.51	3.50 – 31.57		17.27	5.25 – 56.79	
Cause of AKI		0.38			0.38			0.18
Medical	130 (45.8)		0.77	0.43 – 1.38		0.64	0.34 – 1.22	
Surgical /Tr.	22 (39.3)		1.00	–		1.00	–	
Diabetes		0.50			0.50			0.85
Yes	30 (41.1)		0.84	0.48 – 1.41		0.94	0.52 – 1.71	
No	122 (45.5)		1.00	–		1.00	–	
CKD		0.53			0.53			0.040
Yes	49 (47.1)		1.16	0.73 – 1.84		0.54	0.30 – 0.97	
No	103 (43.5)		1.00	–		1.00		
HTN		0.89	1.03		0.89			0.46
Yes	84 (44.9)		1.00	0.67 – 1.58		1.20	0.74 – 1.94	
No	68 (44.2)			–		1.00	–	
Heart failure		0.85			0.85			0.80
Yes	22 (45.8)		1.06	0.58 – 1.96		1.09	0.56 – 2.14	
No	130 (44.4)		1.00			1.00		

^a^test for linear trend

**Table 4:** Occurrence of primary outcomes according to attainment of quality criteria in 341 patients with CA-AKI

Three quality criteria attained	All	Yes	No	*P* value
Patients (%)	341 (100)	152 (44.6)	189 (55.4)	
Outcomes				
Length of stay (days; median and IQR)	10 (4–27)	14 (7–32)	8 (3–20)	0.001
1-yr hosp. Days (days; median and IQR)	23 (7–56)	28 (11–59)	18 (5–46)	0.004
Need for renal replacement therapy (%)	10 (2.9)	6 (3.9)	4 (2.1)	0.33
ICU stay (%)	49 (14.4)	25 (16.4)	24 (12.7)	0.33
1-yr mortality (%)	88 (25.8)	44 (28.9)	44 (23.3)	0.24
^a^Last FU Mean P-Creatinine (μmol/L) ± SD	140 + 119	149 ± 136	133 + 79	0.22
^a^Last FU eGFR (ml/mn) ± SD	54.3 ± 27.4	53.3 ± 29.2	55.2 ± 25.9	0.55
^a^Recovery of renal function (%)	204 (65.2)	96 (66.2)	108 (64.3)	0.72

^a^Values analysed in patients with a minimum 28–day follow–up (*n* = 313 of whom 145 fulfilled the quality criteria and 168 did not)

**Table 5:** Outcomes according to attainment of quality criteria

	Three quality criteria attained vs. no criteria	
Outcomes	Unadjusted HR (95% CI)	Adjusted^a^ HR (95% CI)
1-yr mortality	1.26 (0.83 – 1.91)	1.07 (0.68 – 1.69)
	Unadjusted OR (95% CI)	Adjusted^a^ OR (95% CI)
Renal recovery^b^	1.09 (0.72 – 1.74)	1.19 (0.71 – 1.99)
	Unadjusted additional days (95% CI)	Adjusted additional days (95% CI)
Length of stay	4.1 (−2.4 – 10.7)	1.7 (−5.3 – 8.7)
1-yr hosp. Days	10.5 (−1.2 – 22.2)	6.2 (−5.7 – 18.5)

Survival analyzed by proportional hazards model, renal recovery by logistic regression, length of stay and hospital days by linear regression

^a^Adjusted for age, gender, AKI staging, presence of diabetes, CKI and heart failure

^b^Values analysed in patients with a minimum 28-day follow-up (*n* = 313)

**Fig. 1:**
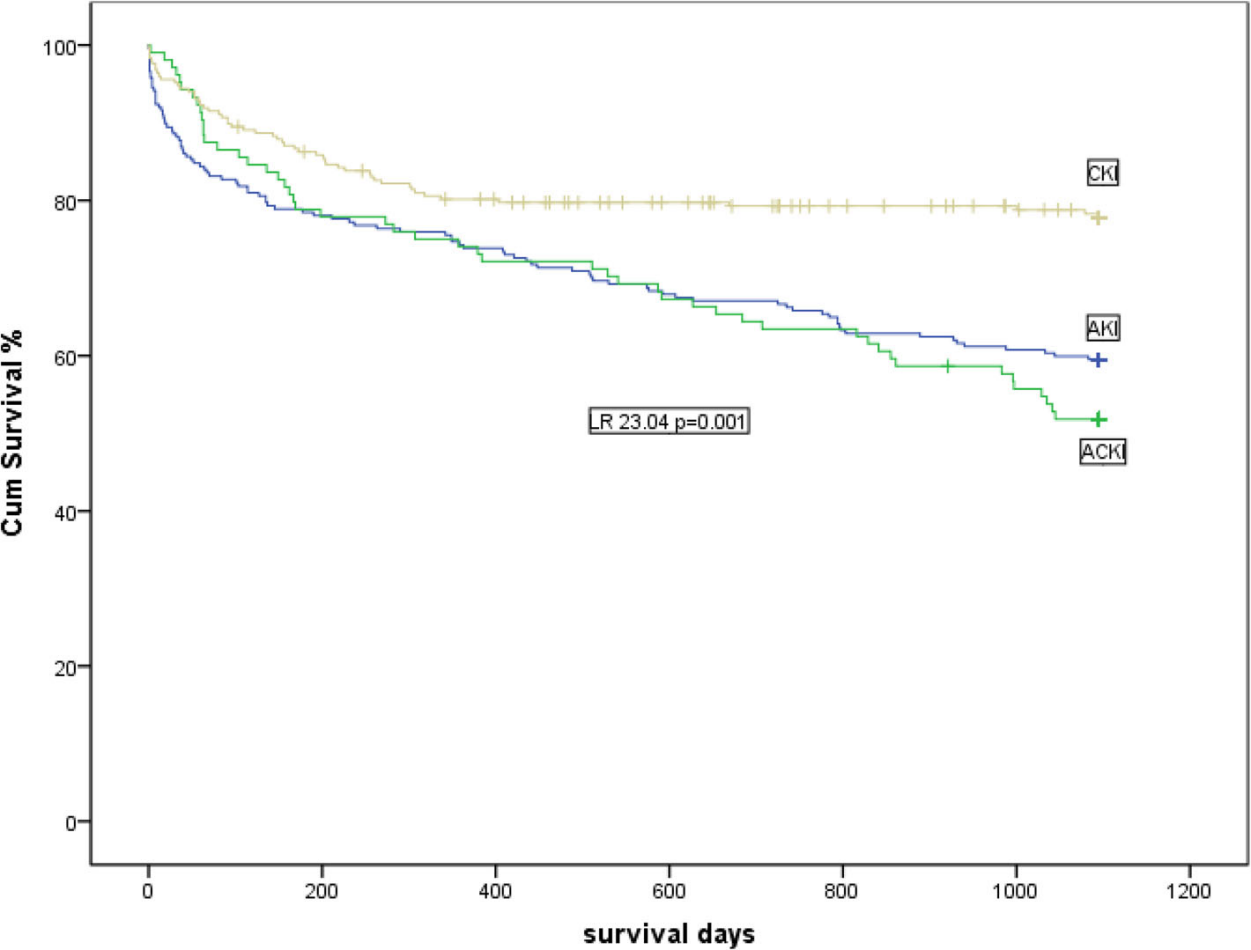
Kaplan-Meier analysis comparing survival in patients with stable CKI, CA-AKI only and CA-ACKI

Three-year mortality in CKI and CA-ACKI patients was 21 and 48%, and in patients with only CA-AKI 40% (*p* < 0.001) (Fig. 1). Mortality was significantly associated with increasing age (OR: 1.04, 95% CI: 1.01-1.06, *p* = 0.001), hypertension (OR: 1.80, 95% CI: 1.01-3.20, *p* = 0.045), neoplasia (OR: 2.41, 95% CI: 1.07–5.45, *p* = 0.034) and ischemic heart disease (OR: 2.70, 95% CI: 1.57–4.64, *p* = 0.001). Compared to patients with stable CKI on admission, CA-AKI, either alone or superimposed on CKI remained significantly associated with three-year mortality in multivariate analysis (Table 6). Renal function could be ascertained in the 299 patients (47%) who had at least one-year follow-up (mean follow-up of 117 ± 33 weeks). Renal function decline was observed in one third of the patients and was significantly associated with age (OR: 1.04, 95% CI: 1.01–1.07, *p* = 0.001) and male gender (OR: 2.18, 95% CI: 1.22–3.90, *p* = 0.008). Compared to CKI patients, those with only CA-AKI had less decrease of renal function (Table 6).

**Table 6:** Three years survival analyzed by proportional hazards model and renal function decline by logistic regression in patients who survived ≥365 days (*n* = 447)

	Three year mortality	
	Unadjusted HR (95% CI)	Adjusted^a^ HR (95% CI)
Chronic kidney disease	1 (reference)	1 (reference)
CA-ACKI	7.47 (2.92 – 19.10) *P* < 0.001	10.85 (4.09 – 28.78) *P* < 0.001
CA-AKI	11.96 (4.55 – 31.46) *P* < 0.001	10.38 (3.98 – 27.04) *P* < 0.001
	Renal function decline (*n* = 299)	
	Unadjusted OR (95% CI)	Adjusted^a^ OR (95% CI)
Chronic kidney disease	1 (reference)	1 (reference)
CA-ACKI	0.82 (0.44 – 1.51) *P* = 0.52	0.68 (0.36 – 1.32) *P* = 0.26
CA-AKI	0.37 (0.21 – 0.64) *P* < 0.001	0.42 (0.23 – 0.75) *P* = 0.003

^a^Adjusted for age, gender, diabetes, HTN, cardiac insufficiency, coronaropathy, neoplasia, infection, CKD, AKI

## Discussion

Patients with a CA-AKI represented 4% of all admissions to the ED during the 8-weeks period of our study. Most of these patients were elderly, with major comorbidities and potentially nephrotoxic medications. CA-AKI in our population was predominantly prerenal and K-DIGO stage 1 at presentation.

Characteristics of our patients were similar to those of the patients described in two previous studies performed in industrialized countries such as the Unites States or the UK [7, 8]. The main differences with the latter study were higher CA-AKI prevalence and severity and the mortality rate at 14 months (45%). A delayed access to the ED leading to a belated diagnosis of AKI and a different patient’s socio-economic profile might explain such differences with a more densely urbanized city such as Geneva.

A high percentage of our patients had potentially nephrotoxic drugs (46% ARBs and/or ACEIs, 37% diuretics, NSAIDs 6%). In CKI patients, NSAIDs were uncommonly used, which reflect likely awareness by their physicians of the deleterious effects of these compounds on renal function. Antibiotics were present also in a minority of our patients and this low number precluded us to infer their responsibility in CA-AKI. Regarding diuretics, there are surprisingly few studies which focus on their role in inducing AKI, especially when they are combined with renin-angiotensin system (RAS) blockers [15]. Fifty-two percent of our patients with AKI had diuretics and they were associated (mainly in fixed dose combinations) with RAS blockers in one quarter of the patients with CA-AKI. Indeed, nearly one half of our patients were either treated by ACEIs or ARBs. This reflects the trend to prescribe these medications in patients with CKI and follows current guidelines which endorse RAS blockers as the cornerstone for nephroprotection. In this study, regarding the occurrence of CA-AKI, only diuretics were found to be detrimental in our population in multivariate analysis. No difference was found between use of ACEis and ARBs in terms of impact on outcome, contrary to the results of recent publications which incriminate both ACEIs and ARBS with renal impairment [16, 17].

For many decades, male gender was recognized as a risk factor for development of CKI and AKI as well. There are multiple explanations, ranging from a potentially more harmful lifestyle, a genetic predisposition to CKI, to an increased rate of post-renal impairment due to prostate hypertrophy [5, 6].

To the best of our knowledge, no studies have examined the impact of early recognition of CA-AKI, its diagnosis work-up and early treatment intervention on hospital outcomes. Only 53% of our patients presenting with CA-AKI were identified as having impaired kidney function. An adequate work-up was performed in 57% of them and ED early intervention in 73%. The discrepancy between recognition and intervention rates can be explained by the high percentage of a prerenal etiology in our study. Although prerenal AKI was probably recognized and fluid replacement prescribed by ED physicians, its severity might not always have been considered as a serious problem worth mentioning in the medical file. Pre-defined quality criteria for management were reached in fewer patients with underlying CKI. This may reflect a tendency in our ED residents to overlook a mild CA-ACKI. Adequate CA-AKI management had no effect on outcomes such as mortality and was unexpectedly associated with a higher length of hospital stay and more hospitalization days within the subsequent year. This unexpected result may reflect either a more severe kidney injury in the adequate AKI management group or delayed discharge of these patients by physicians waiting for recovery of renal function, although renal recovery was not higher in the adequate AKI management group. AKI management may occur too late in patients with CA-AKI to be really effective or a delayed intervention within the ED may not impact outcomes in patients hospitalized after being admitted to the ED.

Regarding long-term outcomes, 3-year mortality was significantly associated in patients with increasing age, coronary heart disease, neoplasia and CA-AKI. Renal function decline was found to be less pronounced in patients with primary CA-AKI, compared to those with previous CKI and CA-ACKI. Outcomes of 130 patients with CA-AKI and prospectively followed for 5 years were recently published that showed that CA-AKI doubled the risk of death and was associated with a 5.7 fold increase in the risk of reaching stage 3 CKI, as compared to 312 patients with normal renal function [18]. Transient azotemia in this study also increased by 2.4 fold the risk of reaching stage 3 CKI. Our results were similar to these data in terms of mortality but differed regarding development of CKI. The older age of our patients and subsequently their increased mortality, their shorter follow-up, the lack of comparison with patients with normal renal function on admission, may explain this apparent discrepancy. Moreover, our CKI patients admitted in the ED were slightly older than those with CA-AKI only. CKD and increasing age are intrinsically linked and the GFR decline is explained by the physiological senescence of kidneys [19]. Severity of CA-AKI was also more pronounced in our CKI patients of whom 57% had stage II/III AKI versus 21% in patients with CA-AKI without previous CKI.

A retrospective analysis from a cohort of US male veterans compared 158 HA- and 560 CA-AKI and found that the latter was 3.5 times more frequent but had similar outcomes to HA-AKI with identical requirements of RRT and an in-hospital mortality of 19.6% [20]. Regarding the specific long-term mortality risk increase in CKI patients after an episode of AKI, this has mainly been studied in patients with HA-AKI [21]. There are few studies which assess the long-term outcome of CKI patients with CA-AKI. In a retrospective UK study, short- and long-term outcomes of 230 patients with CA-AKI (of whom 38% had preexisting CKI disease) compared to patients matched for age and gender without CA-AKI gave a 3-month and 3-year mortality of 16.5% and 45%, respectively [22]. Three-year mortality was not related to the progression of CKI and was increased in those with CA-ACKI. Renal function decline at 3 years was more frequent in this study in patients with CA-ACKI (83%) than in patients with primary CA-AKI (49%). Differences in study design likely explain these discrepancies with our results.

Our prospective data reflect what has previously been described, predominantly in retrospective studies. These studies mostly concern the western world and differ notably from data obtained in less industrialized countries. In these latter reports, demographics of patients with CA-AKI, its severity and short-term mortality do not match our data, although the lack of studies coming from these countries preclude any firm conclusion. For example, mortality rate was 11% in a Indian population with community-acquired AKI, but patients had a mean age of 40 years and etiologies were mainly due to preventable diseases; this epidemiology may have changed over the last 25 years [23]. An observational Brazilian study comparing the differences in patients with community-, hospital- and ICU-acquired AKI underlined the important differences in outcomes based on its etiology [24]. In this study, only 2.2% of the patients had CA-AKI of pre-renal origin, with a 23% in-hospital mortality. Overall need of RRT was 68% in the whole population, which reflects a different case-mix of patients with CA-AKI when compared to our population. Our results reflect typical patients in western societies admitted in the ED with CA-AKI and do not apply to other populations.

Limitations of our study include its observational design, its small size and monocentric nature. Furthermore, we relied on medical files data recorded by ED physicians for the management of AKI which could have biased the quality criteria classification and analysis of outcomes. We only studied patients with a GFR < 60 ml/mn/1.73 m^2^ and therefore may have missed some CA-AKI within the range 90 to 60 ml/mn/1.73 m^2^. By recording the dosage of RAS inhibitors and diuretics, we tried to exclude a different impact on the occurrence of CA-AKI due to a higher dosage of one class of RAS inhibitors. Our follow-up, though complete, is short and most of the episodes of CA-AKI were mild. Although we were able to analyze the short-term recovery of renal function in 93% of our patients, we had data in only 47% of our patients who had a minimum follow-up of 1 year. Indeed, it has been shown that most patients presenting an episode of AKI only stabilize their renal function 1 year after the episode [25].

Strengths of our study are its prospective design, the analysis of the impact of early AKI management on the outcomes, the precise quantification of RABs and diuretics use in CKI and CA-ACKI patients and the 3 year follow-up.

## Conclusions

Our results show that CA-AKI in developed countries is not identified properly in nearly half of cases within the ED of an academic hospital, is frequently found in the elderly and its main etiology is prerenal origin. Medications such as RAS blockers and diuretics are frequently encountered, especially in patients with CKI disease. Although, early adequate AKI management does not seem to significantly change short-term outcomes such as mortality, renal recovery and length of stay, this has to be further studied in larger prospective studies.

## Additional file

Additional file 1: Classification of ARBs, ACEIs and diuretics doses. (DOCX 13 kb)

## Abbreviations

ACEi: Angiotensin-converting enzyme inhibitors
AKI: Acute kidney injury
ARBs: Angiotensin-receptor blockers
CA-ACKI: Community-acquired acute on chronic kidney injury
CA-AKI: Community-acquired acute kidney injury
CKI: Chronic kidney injury
ED: Emergency Department
HA-AKI: Hospital-acquired acute kidney injury
ICU: Intensive care unit
KDIGO: Kidney Disease Improving Global outcomes
NSAIDs: Non-steroidal anti-inflammatory drugs
RAS: Renin-angiotensin system
RRT: Renal replacement therapy
