# Supplementary material for: Risk factors for community-acquired acute kidney injury in patients with and without chronic kidney injury and impact of its initial management on prognosis: a prospective observational study
Source: BMC Nephrol. 2017 Dec 29;18:380. doi: 10.1186/s12882-017-0792-2 (PMC5747946; doi:10.1186/s12882-017-0792-2)
Supplement: Additional file 1: — Classification of ARBs, ACEIs and diuretics doses. (DOCX 13 kb) [file 12882_2017_792_MOESM1_ESM.docx]

**ADDITIONAL FILE 1: TABLE 1.**

**Title:Angiotensin Receptor Blockers, ACE inhibitors and Diuretics dose comparison.**

- ARBs low daily doses: losartan 50 mg, candesartan 4 mg, irbesartan 75 valsartan 40, telmisartan 20 mg, eprosartan 400 mg, olmesartan 20 mg.
- ARBs medium-high daily doses: losartan >50 mg, candesartan >4 mg, irbesartan >75 mg, valsartan >40 mg, telmisartan >20 mg, eprosartan >400 mg, olmesartan > 20 mg
- ACEIs low daily doses: captopril < 6.25 mg tid, enalaparil <5 mg, fosinopril < 5 mg, lisinopril <5 mg, perindopril < 5 mg, ramipril < 2.5 mg, quinapril < 10 mg, trandolapril <1 mg.
- ACEIs medium-high daily doses: captopril >6.25 mg tid, enalaparil >5 mg, fosinopril >5 mg, lisinopril >5 mg, perindopril > 5 mg, ramipril >2.5 mg, quinapril > 10 mg, trandolapril >1 mg.
- Diuretics low daily doses: HCTZ < 25 mg, furosemide < 40mg, torasemide <10 mg, aldactone <25 mg.
- Diuretics medium-high daily doses: HCTZ > 25 mg, furosemide > 40mg, torasemide >10 mg, aldactone >25 mg.
